# A cross-sectional survey exploring the attitude, knowledge, and use of anesthesia teams toward evidence-based practice in Riyadh Saudi Arabia

**DOI:** 10.3389/fpubh.2022.1017106

**Published:** 2022-10-31

**Authors:** Salem Khalaf Al Anazi, Waleed Abdullah Al Zahrani, Mohammed Abdulaziz Alsanad, Matar Saeed Alzahrani, Ibrahim Saeed Al Ghamdi, Abdulmueen Awadh Alotaibi, Mohammed Ali Al maliki, Hamzah Mohammed Asiri, Ghirman Mohammed Alshehri, Abdullah Salem Alanazi, Abdulelah Khalaf Al Anazi

**Affiliations:** ^1^Anesthesia Technology Department, Prince Sultan Military College of Health Sciences, Dhahran, Saudi Arabia; ^2^College of Applied Medical Sciences, King Saud bin Abdulaziz University for Health Sciences, Jeddah, Saudi Arabia; ^3^King Fahad Armed Forces Hospital, Jeddah, Saudi Arabia; ^4^College of Applied Sciences, AlMaarefa University, Riyadh, Saudi Arabia; ^5^Prince Mohammed Bin Abdulaziz Hospital, Riyadh, Saudi Arabia; ^6^Armed Forces Hospital Southern Region, Khamis Mushayt, Saudi Arabia; ^7^King Faisal Specialist Hospital and Research Centre, Riyadh, Saudi Arabia; ^8^Prince Sultan Military Medical City, Riyadh, Saudi Arabia; ^9^Department of Radiology, King Saud Medical City, Riyadh, Saudi Arabia

**Keywords:** evidence based practice, attitudes, use, knowledge, anesthesia teams, Healthcare Professionals

## Abstract

**Background:**

Evidence-based practice (EBP) plays a crucial role in improving the quality of healthcare services by ensuring the delivery of the highest and safest level of patient care since EBP helps in justifying treatment choices to patients. Studies that examine the levels of EBP knowledge, attitudes toward EBP, and use of the use of EBP within anesthetic teams' practice are lacking, hence it is necessary to explore this.

**Aim:**

To evaluate anesthesia teams' levels of knowledge, attitude toward and use of the evidence-based practice in a local hospital in Saudi Arabia.

**Method:**

In one hospital, a cross-sectional survey was conducted using a convenience sampling technique using a validated questionnaire instrument called the Evidence-Based Practice EBP Questionnaire. The questionnaire was distributed through an online method to 173 participants. Descriptive and inferential statistics Tests were utilized to analyse the retrieved data using the SPSS program.

**Results:**

One hundred and forty questionnaires were completed and returned, yielding a response rate of 80.9%. Overall, anesthesia teams showed a high positive attitude toward EBP but low levels of knowledge and use of EBP. Participants with higher levels of education and/or work experience exhibited significantly higher levels of knowledge and use of EBP than those who had lower education levels and/or work experience. Also, higher levels of education and/or work experience exhibited a significant positive association toward a higher level of knowledge and use of EBP. However, attitude levels toward EBP did not exhibit either significant or associated. Physicians showed significantly higher knowledge and use of EBP than non-physicians. Lack of knowledge and lack of time due to workload were the leading barriers encountered by anesthesia teams ATs.

**Conclusion:**

Education level, work experience and job position affect the knowledge, attitude, and use of EBP. Continuous education and minimizing barriers are recommended to enhance the knowledge, attitude, and use of EBP among anesthesia teams in Saudi Arabia.

## Background

In the past, Healthcare Professionals (HCP) based their medical judgments on daily practice, expert sources, or textbooks, and not on scientific evidence-based studies (1). However, in the early 21^st^ century, the picture changed totally due to the huge increase in the quantity of clinical research evidence available (2). Annually about 2.5 million papers are published (3). However, the increase in the quantity of clinical research evidence does not automatically translate into improved patient treatment and care (4). Moreover, rapid changes in the methods of treatments and technologies within the last two decades have made it difficult for HCP to ensure that the available evidence has sufficiently high validity to be implemented into their clinical practice (5). Therefore, EBP exists to play the main role in providing the practitioner with the essential skills to be able to distinguish whether the available evidence is trustworthy (6).

Evidence-based practice is outlined as the process of decision-making by integrating the finest scientific research evidence with clinical experience and the patient's preferences and values (7). The principle of EBP comprises five phases: it starts with formulating a clinical question, searching for the best evidence that answers the question formulated, critically appraising the evidence retrieved to assess its validity and reliability, and implementing the findings with HCPs' expertise and patients' preferences, and finally, evaluating the entire process with the findings of patients' outcomes (8). EBP aims to deliver the most efficient healthcare service where HCPs' decisions are made according to the evaluation of the best evidence rather than depending on traditional treatments (9). Hence, EBP is considered crucial to delivering safe practice with high-quality care to enhance patient outcomes (10).

In the past three decades, EBP has become a global concern for HCPs and administrative staff as well as researchers and policymakers, since it is found to be effective in reducing costs without affecting the quality of care (11). Most healthcare organizations globally have found that EBP offers remarkable outcomes in terms of reducing mortality, morbidity, and medical errors while improving cost-effectiveness (12). Healthcare institutions globally could reduce their expenses by 30% if patients received evidence-based care (13). The World Health Organization (WHO) revealed that employing EBP in daily practice can save a minimum of £4.5 million annually in each hospital (14). In the United Kingdom (UK), EBP reduced around 63% of the total cost of a surgical procedure for carpal tunnel syndrome, which is among the most common surgical procedures performed (15), while in the US, EBP has increased the recovery rate by 30% and saved about 35% of the total cost of treatment for oncology patients suffering from lung cancer, which is the second most common disease diagnosed in the United State (US) (16–18). Accordingly, both national and international healthcare organizations have highlighted the significance of evidence-based care to ensure that patients receive appropriate, high-quality care (19).

Unfortunately, although EBP has proved its effectiveness in different clinical organizations, HCPs' uptake is below optimum levels (14). The gap between the amount of research evidence that exists and the application of such evidence in clinical practice is huge (19). In anesthesia, 30% of clinical decisions taken by ATs have been found to be either unnecessary or potentially harmful to patients (20). For instance, 40% of anesthetists are found to perform the cricoid pressure technique while intubating patients undergoing either emergency or cesarean section procedures, although this technique has been shown to cause esophageal rupture (21). Also, around 80% of medication error events are avoidable if the anesthesia practitioner applied actual evidence-based care (22).

Practitioners have reported encountering several obstacles with the use of EBP in clinical practice (23). Many studies have found that insufficient knowledge about EBP, lack of time due to workload, level of academic qualifications and level of work experience were the main barriers to the use of EBP during clinical practice (24–28). Healthcare organizations also found that HCPs' attitude toward EBP plays a fundamental role in the use of EBP principles in clinical practice (29). This is critical, since applying evidence to clinical practice can take a decade, which could delay the provision of a high-quality healthcare service (30).

To date, limited studies have explored the knowledge, attitudes, and use of EBP among Anesthesia Teams (AT) worldwide. Unfortunately, no studies have investigated these aspects among ATs in Saudi Arabia (SA). Thus, the presence of this gap highlights the need to establish at least a baseline measurement to provide a clear picture to establish strategies to address any future problems that may appear. The study findings would thus underline the knowledge, attitude, and use of EBP among ATs in their respective practices. This could also lead to further research into potential methods for implementing EBP in SA. Hence, the current study aimed to explore the level of knowledge, attitude, and use of evidence-based practice among anesthesia teams in a single hospital in SA. Also, an assessment was performed to investigate the contributing factors that affect the level of knowledge, attitude, and use of EBP within anesthesia teams' professional practice.

## Methods

### Study design

This was a descriptive quantitative cross-sectional survey design. The questionnaire link was sent to the participants' emails *via* an accredited and secured web page platform (SurveyMonkey) to anesthesia staff.

### Target population

The target population was 173 (all anaesthesiologists and anesthesia technologists/technicians who are officially registered in the Saudi Commission for Health Specialities and currently working in Prince Sultan Military Medical City Riyadh Saudi Arabia). A gatekeeper who has access to the contacts of the registered anesthesia staff was utilized to identify the eligible population. Since the data collection period was for 4 weeks between January and February 2020. An auto-reminder was sent asking participants to complete the questionnaire 2 weeks after sending the first link to improve the response rate.

### Sampling

This study used a non-probability convenience sampling method since it reaches all eligible participants who are available and accessible to participants. Importantly, a power calculation was utilized to calculate the needed sample size, to ensure that the sample of the current study would be representative of all anesthesia practitioners in the targeted hospital. The G^*^power programme (version 3.1.9.2) was utilized with a significant value of *p* ≤ 0.05, power of 0.95 and medium effect size of 0.3. This calculation revealed that a sample size of 138 participants was required.

### Data collection

Data were collected by using the Evidence-Based Practice Questionnaire (EBPQ) tool which is considered a valid and reliable self-reported questionnaire (31). The EBPQ comprises closed-ended questions within three subscales: knowledge about, attitude toward, and use of EBP.

Each item of the EBPQ takes the form of a seven-point Likert scale ranging from (1 to 7), and the participants were asked to choose where they found themselves between these seven points. For the knowledge subscale, a score of (1) indicated “Poor,” whereas a score of (7) indicated “Best,” while for the use subscale, a score of (1) indicated “Never” whereas a score of (7) indicated “Frequently.”

However, in the attitude subscale, the participants were asked to indicate where they found themselves between two opposite statements (for instance, “I resent having my clinical practice questioned” to “I welcome questions on my practice”): a score of number (1) means the statement is negative and a score of (7) means the statement is positive. The respondents were asked to indicate their level for each subscale, where (1) indicated the lowest score and (7) indicated the highest. Subsequently, the average score was calculated to determine the level of each subscale. Higher scores indicated higher use of knowledge about and a more positive attitude toward EBP.

In the fourth section, six demographic questions were added to the questionnaire such as gender, age, specialization, job position, academic qualifications, and work experience. Therefore, the questionnaire had four sections with a total of 30 questions. A pilot study was conducted with eight qualified anesthesia professionals (four anaesthesiologists and four technologists) who are not from the targeted population. Piloting was performed to ensure face validity prior to sending the questionnaire to the target population.

### Data analysis

Both descriptive and inferential statistical approaches were utilized to analyse the data collected for this study. These statistical methods were applied using the Statistical Package for Social Sciences (SPSS) program (version 25). Frequency, percentage, mean, and Standard Deviation (SD) were used for the descriptive statistics, and these were presented in tables and charts to simplify explaining the participants' data. Normality test was used, and the data showed that the data were normally distributed; therefore, parametric statistical tests were used for the study's results. Thereafter, inferential statistical tests were used. Both *t*-test and one-way Analysis of Variance (ANOVA) were utilized. Pearson's Correlation test was used to examine whether associations existed between the participants' responses and the variables.

In this study, the cut-off level was determined as follows: mean scores from 1 to 3 were considered low, whereas a score of 4 was considered moderate and 5–7 was considered high.

### Ethical considerations

Ethical approval was obtained from the targeted hospital, in SA, giving the researcher permission to conduct the study in the anesthesia department. Participants have received an information sheet that contains the aim and objectives of the study. Implied consent was the method for obtaining informed consent for this study, and this was clearly stated and explained to the participants in the information sheet. When the participant presses the “submit” button and the questionnaire has been sent, this acts as informed consent. Also, no names or numbers were requested in the questionnaire to ensure the anonymity and confidentiality of the participants.

Ethical approval project code: 1233, 31 Jul Series 2019.

## Results

### Response rate

Out of 173 questionnaires sent, a total of 140 questionnaires were completed presenting a response rate of 80.9%. Of 140 questionnaires, 64 (45.7%) of participants were between 20 and 30 years old, 54 (38.6%) were aged between 31 and 40 years, and 22 (15.7%) were aged 41 or above. The majority of respondents 49 (35%) held diplomas, while 44 (31.5%) held bachelor's degrees, 27 (19.2%) held PhD degrees and finally, 20 (14.3%) held master's degrees. Regarding specialization, most of the respondents were technologists 93 (66.4%), while 47 (33.6%) were anesthetists (Table 1).

**Table 1:** Sample distribution according to demographic characteristics (*n* = 140).

**Variables**	**Variable category**	**Frequency**	**%**
Gender	Male	120	85.7%
	Female	20	14.3%
Age	20–30 years	64	45.7%
	31–40 years	54	38.6%
	41 or more	22	15.7%
Academic qualification	PhD.	27	19.2%
	Master's degree	20	14.3%
	Bachelor's degree	44	31.5%
	Diploma	49	35%
Specialization	Anesthetist	47	33.6%
	Technologist	93	66.4%
Work experience	6 months−10 Years	78	55.7%
	11–20 Years	45	32.1%
	21 years or above	17	12.2%
Job position	Consultant	16	11.4%
	Specialist	20	14.3%
	Resident	11	7.9%
	Senior In-Charge	26	18.6%
	Senior	34	24.2%
	Junior	33	23.6%

Furthermore, more than half of the respondents 78 (55.7%) had between 6 months and 10 years of experience, while 45 (32.1%) had between 11 and 20 years, and only 17 (12.2%) had 21 or more years of experience. As for the job position variable, the majority of participants were in senior positions 34 (24.2%), followed by 33 (23.6%) who were in junior positions, while 26 (18.6%) were senior in charge, 20 (14.3%) were specialists, 16 (11.4%) were consultants and 11 (7.9%) were residents. The majority of respondents were male (85.7%), while 20 (14.2%) were female, and PhD and master's holders were all Anesthetists (Table 1).

### Knowledge of EBP

Overall, ATs revealed a low level of knowledge about EBP, with a mean score of (3.77) out of 7. In this context, participants show a moderate level of knowledge toward sharing and disseminating new ideas about care with colleagues, with a mean score of (4.92; 4.52) respectively. However, ATs' research skills and ability to critically analyse the evidence against set standards were the lowest among the questionnaire items, with an average score of (2.83; 2.91) respectively (Table 2).

**Table 2:** Knowledge, attitude, and use subscales scores (*n* = 140).

**Item**	**Score (mean ±SD)**	**Ranking**
Knowledge subscale		
Sharing of ideas and information with colleagues	(4.92 ± 1.39)	1
Dissemination of new ideas about care for colleagues	(4.80 ± 1.32)	2
IT skills	(4.52 ± 1.50)	3
Ability to review your own practice	(4.07 ± 1.56)	4
Ability to identify gaps in your professional practice	(4.02 ± 1.55)	5
Monitoring and reviewing practice skills	(3.93 ± 1.45)	6
Awareness of major information types and sources	(3.82 ± 1.54)	7
Ability to apply information to individual cases	(3.73 ± 1.76)	8
Converting your information needs into a research question	(3.61 ± 1.54)	9
Knowledge of how to retrieve evidence	(3.53 ± 1.81)	10
Ability to determine how useful (clinically applicable) the material is	(3.11 ± 1.83)	11
Ability to determine how valid (close to the truth) the material is	(3.04 ± 1.80)	12
Ability to analyse critically evidence against set standards	(2.91 ± 1.81)	13
Research skills	(2.83 ± 1.82)	14
Attitudes subscale		
(−) Evidence-based practice is a waste of time (+) Evidence-based practice is fundamental to professional practice	(6.79 ± 1.33)	1
(−) I resent having my clinical practice questioned (+) I welcome questions about my practice	(5.64 ± 1.51)	2
(−) I stick to tried and trusted methods rather than changing to anything new (+) My practice has changed because of evidence I have found	(4.74 ± 2.0)	3
(−) My workload is too great for me to keep up to date with all the new evidence (+) New evidence is so important that I make the time in my work schedule	(3.48 ± 2.09)	4
Use subscale		
Shared this information with colleagues.	(5.11 ± 1.38)	1
Evaluated the outcomes of your practice.	(3.63 ± 2.01)	2
Integrated the evidence you have found with your expertise.	(3.51 ± 1.65)	3
Formulated a clearly answerable question as the beginning of the process toward filling this gap.	(3.27 ± 1.57)	4
Tracked down the relevant evidence once you have formulated the question.	(3.20 ± 1.60)	5
Critically appraised, against set criteria, any literature you have discovered.	(2.92 ± 1.69)	6

### Attitude toward EBP

ATs exhibited a high positive attitude toward EBP, with a mean score of (5.16) out of 7. The participants show a high positive attitude toward the importance of EBP to clinical practice, with a mean score of (6.79). However, ATs show a negative attitude level in “workload is too great for me to keep up to date with new evidence,” with a mean score of (3.48) (Table 2).

### Use of EBP

ATs exhibited a low level of use of EBP, with a mean score of (3.60) out of 7. In this context, ATs show a high use level in sharing information with colleagues, with a mean score of (5.11). However, ATs show poorness in critically appraise literature, with a mean score of (2.92) (Table 2).

### Statistically significant results

Within demographic characteristics, anesthetists have higher levels of knowledge, attitude, and use of EBP than technologists. However, only the knowledge and use levels reveal statistically significant differences between anesthetists and technologists (both at *p* = 0.001). For attitude level, no statistically significant results were found between anesthetists and technologists (*p* = 0.092). Likewise, the results show that levels of knowledge and use of EBP significantly increased as work experience increased among ATs (both at *p* = 0.001). However, although the attitude level increased with work experience among ATs, the result did not reveal a statistically significant difference (*p* = 0.249). This indicates that there is convergence in ATs' level of attitude toward EBP depending on the work experience variable. Regarding academic qualifications, the ATs' levels of knowledge, attitude, and use of EBP increased as academic qualifications increased. However, only knowledge and use levels exhibited statistically significant differences (both at *p* = 0.001). Within the results, the study results show that levels of knowledge, attitude and use of EBP increased as job positions increased. Further, the results show that there were statistically significant differences between levels of knowledge and use among ATs (both at *p* = 0.001). However, for attitude level, there were no significant difference *p* = 0.221 (Table 3).

**Table 3:** Statistical differences and correlations for knowledge, attitude, and practice or use levels according to demographic characteristics (*n* = 140).

	**Questionnaire mean score** ±**SD**		
**Characteristics**	**Knowledge**	**Attitudes**	**Use**
**Gender**			
Male	3.98 ± 1.36	5.04 ± 0.99	3.83 ± 1.40
Female	3.90 ± 1.01	4.89 ± 1.11	3.63 ± 1.26
*p-*value	0.819	0.571	0.546
**Age**			
20–30 years	3.72 ± 1.43	4.62 ± 1.13	3.56 ± 1.46
31–40 years	3.96 ± 0.97	4.83 ± 1.04	4.02 ± 1.13
41 or more	4.10 ± 1.07	5.24 ± 0.87	4.28 ± 1.41
*p-*value	0.278	0.351	0.059
**Academic qualification**			
PhD	4.82 ± 0.78	5.25 ± 0.89	4.65 ± 1.04
Master's degree	4.75 ± 0.86	5.10 ± 0.77	4.49 ± 1.10
Bachelor's degree	4.08 ± 1.13	4.91 ± 1.20	3.73 ± 1.05
Diploma	3.08 ± 1.34	4.66 ± 1.24	3.02 ± 1.43
*p-*value	0.001^*^	0.137	0.001^*^
**Specialization**			
Anesthetist	4.24 ± 0.94	5.13 ± 0.86	4.47 ± 1.15
Technologist	3.30 ± 1.39	4.80 ± 1.18	3.46 ± 1.36
*p-*value	0.001^*^	0.092	0.001^*^
**Work experience**			
6 months−10 years	3.60 ± 1.37	4.76 ± 0.96	3.30 ± 1.27
11–20 years	4.19 ± 1.04	4.91 ± 1.20	4.08 ± 1.30
21 years or more	5.04 ± 1.05	5.28 ± 0.84	4.56 ± 1.35
*p-*value	0.001^*^	0.249	0.001^*^
**Job position**			
Consultant	4.87 ± 1.19	5.30 ± 0.75	4.86 ± 0.98
Specialist	4.43 ± 1.20	5.21 ± 0.90	4.27 ± 1.01
Resident	4.35 ± 0.83	5.02 ± 1.38	4.14 ± 0.99
Senior In-charge	4.15 ± 0.47	4.99 ± 1.23	3.87 ± 1.59
Senior	4.07 ± 1.27	4.66 ± 0.68	3.73 ± 1.14
Junior	2.76 ± 1.29	4.60 ± 0.98	2.86 ± 1.16
*p-*value	0.001^*^	0.221	0.001^*^

(SD) Standard Deviation,

(*) statistically significant P < 0.05 level.

### Associations according to demographic characteristics

Results reveal that there were no associations between attitude levels and any of the demographic variables. However, there were positive, statistically significant associations between the level of knowledge about EBP among ATs and academic qualification (*R* = 0.503) and work experience (*R* = 0.363). These findings indicate that ATs' knowledge increased with the increment of academic qualification and/or work experience. Also, there were positive, statistically significant associations between the levels of use of EBP among ATs and academic qualifications (*R* = 0.483), work experience (*R* = 0.199) and job position (*R* = 0.409), indicating that ATs' use of EBP increases in line with each of these variables. Regarding gender, age and specialization, the results show no significant association (Table 4).

**Table 4:** Associations of knowledge, attitude and use levels according to demographic characteristics (*n* = 140).

**Dimensions and associations (R)**		**Demographic characteristics**					
		**Gender**	**Age**	**Academic qualification**	**Specialization**	**Work experience**	**Job position**
Attitudes	*R*	0.048	0.027	0.017	0.014	0.056	0.071
	*p*-value	0.074	0.061	0.082	0.093	0.067	0.094
Knowledge	*R*	0.019	0.040	0.503^**^	0.020	0.363^**^	0.043
	*p*-value	0.081	0.091	0.028	0.061	0.001	0.058
Use	*R*	0.051	0.035	0.483^**^	0.029	0.199^**^	0.409^**^
	*p*-value	0.097	0.088	0.001	0.062	0.024	0.001

(R) Pearson's correlation,

(**) statistically significant correlation.

### Association between the attitude and use subscales toward the knowledge subscale

Results exhibit that there was a significant positive association between the levels of attitude and knowledge toward EBP among ATs (*R* = 0.372, *p* = 0.036), which indicates that the ATs' attitude toward EBP increased with the increment of knowledge level. Similarly, there was a significant positive association between the level of use of EBP among ATs and the level of knowledge (*R* = 0.641, *p* = 0.023), showing that the ATs' use of EBP increased with the increment of knowledge level (Table 5).

**Table 5:** Associations between the attitude and use subscales and the knowledge subscale.

**Subscales**		**Knowledge subscale**
Attitude	r	0.372^**^
	*p*-value	0.036
Use	r	0.641^**^
	*p*-value	0.023

(r) Pearson's correlation,

(**) statistically significant correlation.

## Discussion

The study aimed to investigate the level of ATs' knowledge, attitude, and use of EBP in a large local hospital in SA. The current study revealed that amongst the three subscales, attitude achieved the highest score, followed by knowledge, while the lowest score was for the use of EBP. The current study's findings suggest that ATs overall have a low level of knowledge about EBP. This result was similar to the findings of previous studies (24, 26, 32, 33). However, it contradicts the findings of previous studies (25, 34, 35).

Within the knowledge subscale, the highest items scored by ATs related to sharing and dissemination of evidence to colleagues, whereas the lowest scoring items were in “research skills,” “ability to critically appraise literature,” and “ability to determine how valid the material is” and “ability to determine how useful the material is.” This is interesting since the highest and lowest scoring items were also similarly ranked in previous studies (24–26). The similarities in the results reported by the latter studies indicate that the lack of knowledge could be attributed to the weakness of the teaching approaches in terms of providing sufficient teaching modules to explain the principles of EBP during academic studies. This was clear in the current study's significant difference in the level of knowledge as academic qualifications increased. In this study, participants with diplomas showed the lowest score, while those with PhD degrees had the highest level of knowledge about EBP. This could be because the diploma curriculum for anesthesia practitioners in SA does not contain any research modules to teach students how to read and evaluate research findings (36). The current study also found a significant positive association between level of knowledge and academic qualification, meaning that higher qualifications were related to higher knowledge. This is congruent with the previous studies (33, 35). Importantly, although the latter two studies reported different overall results regarding the level of knowledge about EBP, these studies showed similar results regarding the association between qualifications and level of knowledge. The findings of this study also revealed that work experience exhibited a significant positive association with a high level of knowledge. The increase was comparable with the findings of previous studies (24–26, 32).

Attitude toward EBP was highly positive amongst ATs in this study. This was comparable to previous studies (37–40). This highly positive attitude could be because the targeted hospital undergoes annual evaluation by the Saudi Central Board for Accreditation of Healthcare Institutions (CBAHI) and the Joint Commission (JCI), and these organizations play a main role in enhancing the use of EBP. This high positive attitude indicates that ATs have good use of- the significance of EBP in delivering the highest quality of healthcare. On the other hand, the lowest attitude level reported by ATs was toward the statement “My workload is too great for me to keep up to date with all the new evidence,” which could be considered as a barrier not only to the level of attitude but also toward the use of EBP at the bedside. The presence of this barrier is critical, since attitudinal barriers toward EBP can be more difficult to overcome than knowledge barriers, and thus could affect the application of retrieved evidence in actual practice (41). The current study found a statistically significant positive association between the attitude and knowledge subscales, meaning that attitude level increases as knowledge level increases.

Results from the current study revealed that ATs have low levels of use of EBP. This finding concurred with previous studies (28, 42, 43). However, it contrasted with previous studies (26, 27). This might be because the former three studies and the present study reported a lack of knowledge about EBP. In this study, a significant positive association between the use and knowledge subscales, meaning that level of use of EBP increases with the increase of knowledge. This is interesting since this can be the main influence toward the low use level. Additionally, the current study's results found that the use of EBP among ATs showed a significant increase as their academic qualification increased.

The current study also found that work experience influences the level of use of EBP. The use of EBP increased significantly with higher work experience. This was comparable to the previous studies (28, 42, 43). However, it contradicted the previous studies (26, 27). It could be because HCPs with more than 10 years of experience become more confident in using EBP in clinical practice, whereas those with < 10 years of experience have limited practical knowledge (44). Moreover, the current study found a significant increase in the practice of EBP as the seniority of job positions increases. This result was comparable with the findings of previous studies (28, 43). In fact, the tasks, and duties of anesthesia practitioners in senior positions tend to be more administrative than clinical, allowing more time to search for and evaluate evidence (45). In contrast, residents and juniors have huge workloads, such as covering on-calls duties, attending cardiac arrest events, and visiting patients before and after surgery, which makes the use of EBP in clinical practice difficult (46).

## Limitations

Limitations of the current study could be attributed to the fact that the data collection instrument utilized in the study was a self-reported questionnaire, which might be subject to self-reporting bias. Also, conducting a cross-sectional study in a single hospital with a relatively small sample size limits the ability to generalize the results to all ATs in SA.

## Conclusion

This study was conducted to investigate ATs' levels of knowledge, attitude, and use of EBP in a single hospital in SA. Generally, the findings show that ATs had poor knowledge and use of EBP. However, ATs showed high positive attitudes toward EBP. Also, the study revealed that certain demographic characteristics influenced the overall results within the three subscales levels. Anesthesia practitioners' academic qualifications, Job positions, and work experience revealed a significant result with a positive correlation higher level of knowledge and use of EBP. In this study, lack of knowledge about EBP principles and lack of time due to workload were reported by ATs as the main barriers that affected their attitude and use levels toward EBP. Therefore, providing training and workshops about research skills, critical appraisal skills and research utilization to all ATs should be implemented, as this would reinforce their knowledge, which in turn would reflect on the high use of EBP in clinical practice.

## Data availability statement

The original contributions presented in the study are included in the article/supplementary material, further inquiries can be directed to the corresponding author.

## Ethics statement

The studies involving human participants were reviewed and approved by Prince Sultan Military Medical City Scientific Research Center. The patients/participants provided their written informed consent to participate in this study.

## Author contributions

All authors listed have made a substantial, direct, and intellectual contribution to the work and approved it for publication.

## Conflict of interest

The authors declare that the research was conducted in the absence of any commercial or financial relationships that could be construed as a potential conflict of interest.

## Publisher's note

All claims expressed in this article are solely those of the authors and do not necessarily represent those of their affiliated organizations, or those of the publisher, the editors and the reviewers. Any product that may be evaluated in this article, or claim that may be made by its manufacturer, is not guaranteed or endorsed by the publisher.
